# Effectiveness of Movement Representation Techniques in Chronic Non-Specific Spinal Pain: A Systematic Review and Meta-Analysis

**DOI:** 10.3390/medicina62081534

**Published:** 2026-08-09

**Authors:** Nuray Alaca, Ali Ömer Acar, Sergen Öztürk, Dilek Çağrı Arslan

**Affiliations:** 1 Department of Physiotherapy and Rehabilitation, Faculty of Health Sciences, Acibadem Mehmet Ali Aydinlar University, Kerem Aydınlar Kampüsü, Icerenkoy Mah. Kayısdagı Cad. No:32, Atasehir, 34752 Istanbul, Türkiye; aliomer.acar@acibadem.edu.tr (A.Ö.A.); sergen.ozturk@acibadem.edu.tr (S.Ö.); dilek.cagri@acibadem.edu.tr (D.Ç.A.); 2 Department of Physiotherapy and Rehabilitation, Institute of Graduate Studies, Biruni University, 34015 Istanbul, Türkiye; 3 Physiotherapy and Rehabilitation Doctorate Programme, Institute of Health Sciences, Marmara University, 34854 Istanbul, Türkiye; 4 Department of Cardiopulmonary Physiotherapy and Rehabilitation, Institute of Health Sciences, Bezmialem Vakif University, Fatih, 34093 Istanbul, Türkiye

**Keywords:** motor imagery, mirror therapy, chronic spinal pain, neck pain, low back pain, meta-analysis, systematic review

## Abstract

*Background and Objectives*: Movement representation techniques (MRTs), including motor imagery and mirror therapy, have been investigated in several chronic pain conditions; however, their effectiveness in chronic non-specific spinal pain (CNSP) has not been specifically synthesized using meta-analytic methods. This systematic review and meta-analysis aimed to evaluate the effects of MRTs on pain intensity, disability, and kinesiophobia in adults with CNSP. *Materials and Methods*: A systematic search was conducted in PubMed, CINAHL, Scopus, Cochrane Library, and PEDro, supplemented by grey literature sources. Randomized and non-randomized controlled trials investigating MRTs as a standalone or adjunct intervention in adults with CNSP were eligible. Methodological quality was assessed using the PEDro scale, risk of bias was evaluated using the RoB 2 tool, and certainty of evidence was evaluated using the GRADE approach. Effect sizes were calculated as Hedges’ g using a random-effects model. The review was registered in PROSPERO (CRD42023488671). *Results*: Nine studies involving 411 participants were included in the systematic review, of which eight randomized trials (337 participants) contributed to the meta-analyses. MRTs showed a large and statistically significant effect on pain intensity (Hedges’ g = −1.63; 95% CI: −2.56 to −0.70; *p* = 0.001) and a moderate statistically significant effect on disability (Hedges’ g = −0.64; 95% CI: −1.03 to −0.26; *p* = 0.001). The effect on kinesiophobia was large but not statistically significant (Hedges’ g = −1.55; 95% CI: −3.25 to 0.16; *p* = 0.075). In analyses restricted to motor imagery, significant effects were observed for pain intensity (Hedges’ g = −1.87; 95% CI: −2.95 to −0.79) and disability (Hedges’ g = −0.75; 95% CI: −1.15 to −0.35), consistent in direction with the primary analyses. Heterogeneity was substantial for pain intensity and kinesiophobia; all pooled trials were judged to be at high risk of bias or to raise some concerns, and the certainty of evidence was very low for all three outcomes. *Conclusions*: Very low-certainty evidence suggests that MRTs may reduce pain intensity and improve disability in adults with CNSP. Given the high risk of bias and substantial heterogeneity, these estimates are exploratory and do not support firm clinical recommendations. Further well-designed randomized controlled trials with standardized MRT protocols and long-term follow-up are needed.

## 1. Introduction

Chronic non-specific spinal pain (CNSP) is a highly prevalent musculoskeletal condition affecting the cervical, thoracic, and lumbar spine and is recognized as one of the leading causes of disability worldwide. As a major contributor to the global burden of musculoskeletal disorders, CNSP substantially impairs quality of life, functional capacity, and sleep, while also generating considerable socioeconomic costs through productivity loss, increased healthcare utilization, and disruption of family and social roles [1]. Among spinal pain subgroups, low back pain demonstrates the highest prevalence (43%), followed by neck pain (32%), whereas thoracic pain is reported less frequently (13%) [2]. In 2020, approximately 619 million people worldwide experienced low back pain and 223 million experienced neck pain [3,4,5]. The lifetime prevalence of low back pain approaches 80%, with 15–40% of individuals developing chronic symptoms following an acute episode [6]. Neck pain similarly affects 30–50% of the population annually, with 14–17% progressing to chronicity [1,7]. Although thoracic spinal pain occurs less frequently, persistent symptoms have been reported in approximately 10–20% of individuals [2,8].

The pathophysiology underlying CNSP reflects a complex interplay of central sensitization, reorganization within sensory–motor networks, and biopsychosocial contributors, while a specific anatomical cause can be identified in only 5–10% of patients [9,10]. Central sensitization refers to an exaggerated responsiveness of the central nervous system to normal or even subthreshold stimuli and involves mechanisms such as disrupted connectivity within the default mode network, impaired descending inhibitory modulation, and increased activity in pain-facilitatory pathways. In this context, altered central pain processing has been demonstrated particularly in individuals with chronic low back pain (CLBP) and chronic neck pain (CNP), and similar mechanisms have been suggested across CNSP conditions [11,12,13]. Furthermore, patients with CNSP exhibit dysfunction in descending nociceptive inhibition and morphological alterations in gray matter, supporting the presence of central nervous system hyperexcitability [14,15,16]. Importantly, emerging evidence indicates that these morphological alterations may be partially reversible following effective treatment interventions [13,17]. These findings suggest that CNSP is not solely a peripheral musculoskeletal disorder but also involves maladaptive neuroplastic changes within central sensorimotor networks. Such neuroplastic alterations may also contribute to disturbances in cortical body representation and sensorimotor integration, which can lead to altered movement perception and impaired motor control in individuals with CNSP [18,19,20]. Consequently, rehabilitation approaches increasingly aim to target central pain mechanisms and cortical reorganization rather than focusing exclusively on peripheral tissues.

Movement Representation Techniques (MRTs), including motor imagery, action observation, mirror therapy, visual mirror feedback, and graded motor imagery, represent rehabilitation strategies designed to activate sensorimotor networks without provoking nociceptive input. Although these techniques differ in their specific modalities—motor imagery engages mental simulation of movement, action observation relies on the mirror neuron system, and mirror therapy manipulates visual feedback to alter perceived body representation—they share a common theoretical foundation: all engage sensorimotor neural networks through mental or observed movement without requiring actual physical execution, thereby activating cortical motor and somatosensory areas while minimizing nociceptive input. Through this shared mechanism, MRTs are thought to facilitate adaptive neuroplasticity and restore altered cortical representations associated with persistent pain conditions [21,22,23,24]. MRTs have been widely applied to induce neuroplastic changes within the central nervous system in several pain-related conditions, including complex regional pain syndrome, phantom limb pain, low back pain, shoulder pain, and post-stroke pain [25,26,27,28,29,30,31,32]. Evidence from systematic reviews and meta-analyses further supports the potential of MRTs to reduce pain intensity and improve sensorimotor function in these populations [21,25,26,30,31,33,34]. However, most of this evidence originates from heterogeneous pain conditions, and the effectiveness of MRTs specifically in individuals with CNSP remains unclear. A recent systematic review by García-Alonso et al. (2026) investigated the effectiveness of MRTs in individuals with craniocervical and orofacial pain, including studies involving CNP [35]. Although this review suggested potential benefits of MRTs on pain sensitivity and cervical sensorimotor function, several included studies were conducted in asymptomatic participants and most interventions consisted of single-session experimental paradigms rather than structured therapeutic rehabilitation programs. Consequently, the clinical effectiveness of MRTs within a broader CNSP population remains insufficiently synthesized.

Given the growing recognition of central mechanisms in CNSP and the theoretical potential of MRTs to modulate cortical processing, a comprehensive synthesis of the available clinical evidence in this population is warranted. Despite increasing interest in these interventions, no systematic review and meta-analysis has specifically evaluated the effectiveness of MRTs in individuals with CNSP. Therefore, the aim of this systematic review and meta-analysis was to investigate the effectiveness of movement representation techniques on pain intensity, disability, and kinesiophobia (fear of movement) in individuals with CNSP, compared with active treatment, sham, or no-treatment control interventions.

## 2. Materials and Methods

### 2.1. Protocol and Registration

This meta-analysis was carried out in full compliance with the Preferred Reporting Items for Systematic Reviews and Meta-Analyses (PRISMA) statement (Supplementary Table S1) [36]. In addition, the review protocol was prospectively registered in the International Prospective Register of Systematic Reviews (PROSPERO) under the registration number CRD42023488671.

### 2.2. Search Strategy

Two researchers (A.Ö.A. and S.Ö.) searched five major databases independently—PubMed, Physiotherapy Evidence Database (PEDro), EBSCO-CINAHL Complete (Cumulative Index to Nursing and Allied Health Literature), Scopus and Cochrane Library. Eligibility was restricted to publications in English or Turkish reflecting the language competencies of the review team and the absence of translation resources. The strategy was first built and refined in PubMed/Medline and validated against the Peer Review of Electronic Search Strategies (PRESS) framework [37]. Medical Subject Headings (MeSH) terms and relevant keywords were selected for both participants and interventions, and the final strategy was formulated in collaboration with a librarian and content experts to ensure methodological rigor. To broaden the scope and minimize publication bias, additional steps were taken. Grey literature sources included the Turkish Council of Higher Education Thesis Center, ProQuest Dissertations & Theses Citation Index, and manual screening of reference lists from relevant systematic reviews and Google Scholar. Only peer-reviewed articles and full-text theses involving adult populations were included. To enhance reliability and reduce selection bias, the screening process was carried out independently by two reviewers (A.Ö.A. and S.Ö.). Full search strategies for each database and grey literature source are provided in Supplementary Table S2. Duplicate records and review articles were identified and removed using Rayyan [38], a free web-based tool (Qatar Computing Research Institute, Doha, Qatar).

### 2.3. Eligibility Criteria

Eligibility was framed with the PICOS framework (Participants, Interventions, Comparisons, Outcomes, and Study design) [39], developed in consultation with a librarian and content experts, which also served to define the objectives of the review.

The components were defined as follows:

- Participants (P): Adults aged 18 years and older with CNSP, including individuals with non-specific low back pain, non-specific neck pain, and non-specific thoracic spinal pain.
- Interventions (I): Movement Representation Techniques (MRTs), including motor imagery, action observation, mirror therapy, visual mirror feedback, and graded motor imagery, delivered either as standalone interventions or as part of a multimodal rehabilitation program.
- Comparisons (C): Control groups receiving no intervention, placebo/sham intervention, or another conservative treatment modality.
- Outcomes (O): Studies assessing at least one of the following outcomes: pain intensity, spinal range of motion (cervical, thoracic, lumbar), disability and kinesiophobia or fear-avoidance beliefs.

Study design (S): Randomized controlled trials and non-randomized controlled trials were eligible for inclusion. Eligible studies were limited to publications in English or Turkish.

### 2.4. Exclusion Criteria

The exclusion criteria were defined as follows:

Studies involving specific or non-musculoskeletal pathologies, including spinal tumors, infections, fractures, systemic inflammatory diseases, referred pain originating from non-spinal sources, neurological disorders, or conditions with clear structural or neurological involvement such as radiculopathy.

Studies involving single-session experimental interventions assessing only immediate effects were excluded because the aim of this review was to evaluate the effectiveness of structured rehabilitation interventions.

Studies for which the full text was unavailable. Authors of studies with inaccessible full texts were contacted; however, studies remained excluded if no response was received or the full text could not be obtained.

### 2.5. Selection Process

Prior to the screening process, all authors independently examined a random sample of 33 titles and abstracts (representing 10% of all identified unique records) to evaluate the applicability of the inclusion and exclusion criteria. The two reviewers responsible for the screening (S.Ö. and D.Ç.A.) demonstrated acceptable inter-rater reliability with the senior author (N.A.), achieving agreement rates of 90.91–93.94% and Cohen’s kappa values ranging from 0.673 to 0.796 [40].

**Figure 1:**
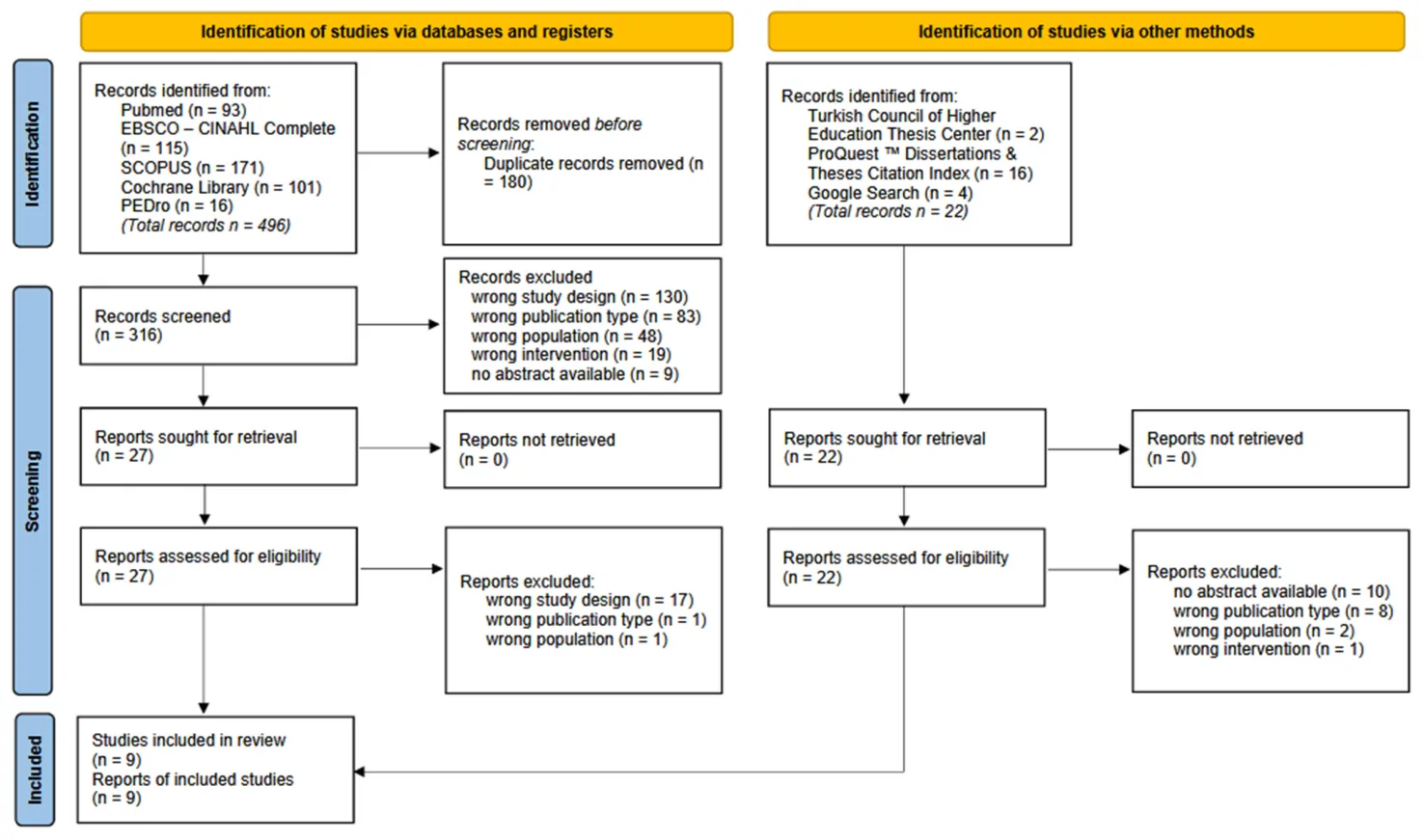
PRISMA 2020 flow diagram for new systematic reviews which included searches of databases, registers and other sources.

Study selection was independently conducted by two reviewers (S.Ö. and D.Ç.A.), both physiotherapists pursuing doctoral studies in musculoskeletal rehabilitation. The reviewers first assessed all titles and abstracts according to the eligibility criteria, followed by full-text evaluation of studies deemed potentially relevant. Inclusion decisions for each study were reached through consensus after independent review by all authors. In cases where consensus was not achieved, the senior author (N.A.) made the final determination. For one study, the authors contacted the original investigator due to missing information or restricted access to the full text (n = 1), and the requested full text and supplementary data were successfully obtained [41]. The flowchart summarizes the screening process and reasons for exclusion (Figure 1).

### 2.6. Data Extraction

Following the study selection process, two researchers (S.Ö. and D.Ç.A.) independently extracted data using a standardized Cochrane data extraction form. Discrepancies were resolved through discussion and consensus with the senior author. Extracted data included study identification, participant characteristics, definitions of chronic pain, symptom-duration thresholds, pain-intensity eligibility criteria, intervention and control groups, assessment time points, outcome measures, and key findings. Continuous outcomes were reported as means and standard deviations. When continuous data were reported as medians and interquartile ranges, these values were converted to means and standard deviations using the methods described by [42,43]. For studies reporting more than one assessment after the intervention, the assessment closest to completion of the intervention was used for quantitative synthesis. When an immediate post-treatment assessment was not available in an extractable group-level form, the earliest available post-intervention follow-up was used. This applied to Nobusako et al. [44], in which the repeated measurements during the intervention were obtained before individual treatment sessions; therefore, the 15-day follow-up was used as the earliest extractable post-intervention assessment.

### 2.7. Methodological Quality and Risk of Bias Assessment

Methodological quality was appraised with the PEDro scale [45], an instrument developed for experimental physiotherapy trials in which ten of the eleven items contribute to a total score out of 10. Scores were interpreted as excellent (9–10), good (6–8), fair (4–5), or poor (below 4) [46,47]. Where a trial had already been rated in the PEDro database (https://www.pedro.org.au/, accessed on 22 April 2026), that rating was adopted; the remaining trials were rated independently by two reviewers (A.Ö.A. and D.Ç.A.), with disagreements settled in discussion with the senior author (N.A.), who was unaware of the initial ratings. Quality scores did not determine eligibility for the systematic review.

Risk of bias was appraised with the Cochrane Risk of Bias 2 (RoB 2) tool [48]. by the same two reviewers working independently. Because the three outcomes differ both in how they are measured and in the biases to which they are susceptible, a separate appraisal was performed for pain intensity, disability, and kinesiophobia. Within each appraisal, the five RoB 2 domains—randomization, departures from intended interventions, missing data, outcome measurement, and selective reporting of results—were each judged low risk, some concerns, or high risk, and these judgements were combined into an overall rating for that outcome [49]. Disagreements between reviewers were resolved through discussion with the senior author (N.A.). RoB 2 is validated for randomized trials only. Trials that did not use true randomization were therefore excluded from all pooled analyses and were not appraised with RoB 2; such trials were retained in the systematic review and are described narratively in results section.

### 2.8. Data Synthesis and Analysis

All statistical analyses were conducted using Comprehensive Meta-Analysis software, Version 3 (CMA V3; Biostat Inc., Englewood, NJ, USA). For continuous outcomes, standardized between-group differences in pre-to-post change were calculated and expressed as Hedges’ g with corresponding variance estimates subsequently entered into Comprehensive Meta-Analysis Version 3. Where parallel measurements represented the same outcome construct in the same participants, they were combined so that each study contributed only one estimate per outcome domain. In Nobusako et al. [44], VAS pain during right and left cervical rotation was therefore combined into a single study-level pain estimate, avoiding both double-counting of participants and arbitrary selection of one movement direction. For multi-arm trials, the comparison that most directly isolated the contribution of MRTs was selected, and each study contributed only one comparison to a given meta-analysis. Accordingly, Javdaneh et al. [50] contributed the neck stabilization exercise plus motor imagery versus neck stabilization exercise comparison, whereas Abdel-Aal et al. [51] contributed the motor imagery plus conventional physiotherapy versus conventional physiotherapy comparison. Given the anticipated clinical and methodological heterogeneity among included studies, random-effects models using the DerSimonian–Laird estimator were applied for all meta-analyses. Between-study heterogeneity was quantified using the I^2^ statistic, with values of 25%, 50%, and 75% representing low, moderate, and high heterogeneity, respectively [49]. To explore whether intervention type contributed to the observed between-study heterogeneity, an exploratory subgroup analysis was performed for pain intensity and disability outcomes. Because motor imagery was the predominant intervention modality among the included studies, subgroup analyses were restricted to studies evaluating motor imagery interventions. Seven of the eight studies included in the pain intensity meta-analysis and six of the seven studies included in the disability meta-analysis used motor imagery. Formal comparisons between different movement representation technique modalities were not feasible because only one study in each analysis evaluated a non-motor imagery intervention. Given the relatively small sample sizes of most included studies, Hedges’ g was selected as the effect size metric. Effect sizes of 0.2, 0.5, and 0.8 were interpreted as small, medium, and large, respectively [52]. Small-study effects were examined both graphically and statistically. Funnel plots were inspected for asymmetry, which was then tested formally with the rank correlation test of Begg and Mazumdar and with Egger’s regression. Where asymmetry was apparent, the trim-and-fill procedure of Duval and Tweedie was used to estimate how many trials might be missing and how their inclusion would shift the pooled estimate [52,53]. Given how few trials were pooled, all of these results were treated cautiously.

### 2.9. Certainty of Evidence Assessment

Certainty of evidence was graded with the Grading of Recommendations Assessment, Development and Evaluation (GRADE) approach, separately for each pooled outcome. Two reviewers (A.Ö.A. and D.Ç.A.) worked independently and reconciled their judgements by consensus. Each body of evidence began at high certainty, as only randomized trials were pooled, and was then examined for risk of bias, inconsistency, indirectness, imprecision, and publication bias. A domain judged serious lowered certainty by one level and a domain judged very serious by two, yielding a final rating of high, moderate, low, or very low. The resulting ratings, together with the pooled estimates, the number of contributing trials and participants, and the reasons for any downgrade, are presented in a Summary of Findings table.

## 3. Results

### 3.1. Study Selection

A total of 496 records were identified through database searches (PubMed, *n* = 93; EBSCO–CINAHL Complete, *n* = 115; Scopus, *n* = 171; Cochrane Library, *n* = 101; PEDro, *n* = 16) and 22 additional records through other sources (Turkish Council of Higher Education Thesis Center, *n* = 2; ProQuest Dissertations & Theses, *n* = 16; Google Search, *n* = 4). Following the removal of 180 duplicate records, 316 records were screened at the title and abstract level, of which 289 were excluded due to wrong study design (n = 130), wrong publication type (n = 83), wrong population (n = 48), wrong intervention (n = 19), or unavailable abstract (n = 9). Full-text assessment was performed for 49 reports (27 from databases, 22 from other sources), and 40 were subsequently excluded. Nine studies met all eligibility criteria and were included in the systematic review; eight randomized controlled trials contributed to the meta-analyses (Figure 1).

### 3.2. Methodological Quality

The methodological quality of the included studies was assessed using the PEDro scale, with scores ranging from 2 to 8 out of 10 (Table 1). Two studies were rated as good methodological quality, scoring 8/10 [51,54]. Six studies demonstrated good methodological quality with PEDro scores ranging from 6 to 7 [41,44,50,55,56,57]. One study was classified as poor methodological quality with a PEDro score of 2/10 [58], primarily due to the absence of randomization, blinding, and allocation concealment procedures. Daskalaki et al. (2024) [58] received a poor PEDro score of 2/10, primarily because of the absence of true randomization and other key methodological safeguards. On closer examination, the study was found to use semi-randomized allocation rather than true randomization and was therefore retained in the systematic review but excluded from all meta-analyses. All pooled estimates therefore derive exclusively from randomized controlled trials.

### 3.3. Study and Participant Characteristics

Nine studies meeting the eligibility criteria were included in this systematic review and their characteristics are summarized Table 2. Studies were published between 2012 and 2025, with a total of 411 participants and sample sizes ranging from 17 to 72. Six studies involved CNP [41,44,50,51,54,57] and three involved CLBP [55,56,58]; no eligible studies on chronic thoracic spinal pain were identified. Chronic pain was generally defined as symptoms lasting ≥3 months or >12 weeks, although two studies used a ≥6-month threshold. Explicit pain-intensity eligibility criteria were reported in three studies. Motor imagery was the most frequently applied MRT, used in seven studies either as a standalone or adjunct intervention [41,50,51,54,56,57,58]. Mirror therapy was employed in one study [55] and a motor imagery-driven gaze direction recognition task in one study [44]. In all but one study, MRT was delivered in combination with exercise or conventional physiotherapy; in one study, MRT was applied as a standalone intervention [44]. Intervention duration ranged from three to ten weeks. Pain intensity and disability were the most commonly reported outcomes; kinesiophobia was assessed in three studies [41,50,56].

### 3.4. Quantitative Synthesis (Meta-Analysis)

#### 3.4.1. Pain Intensity

**Figure 2:**
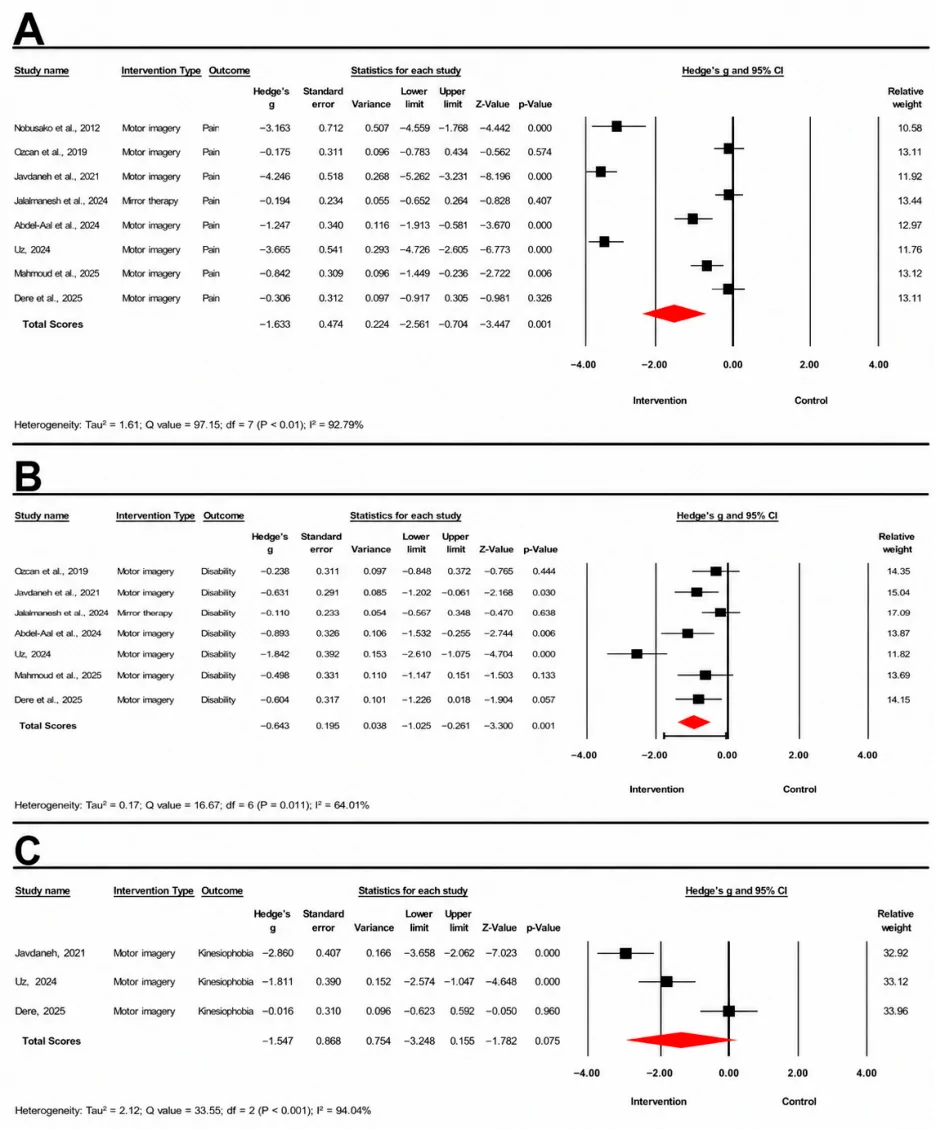
Forest plots showing the effects of movement representation techniques on pain, disability and kinesiophobia in patients with non-specific spinal pain. CI, confidence interval. Negative Hedges’ g values favor the intervention group. Squares indicate individual study effects and diamonds indicate pooled effects. Horizontal lines represent 95% confidence intervals. (**A**): Pain [41,44,50,51,54,55,56,57]; (**B**): Disability [41,50,51,54,55,56,57]; (**C**): Kinesiophobia [41,50,56].

**Figure 3:**
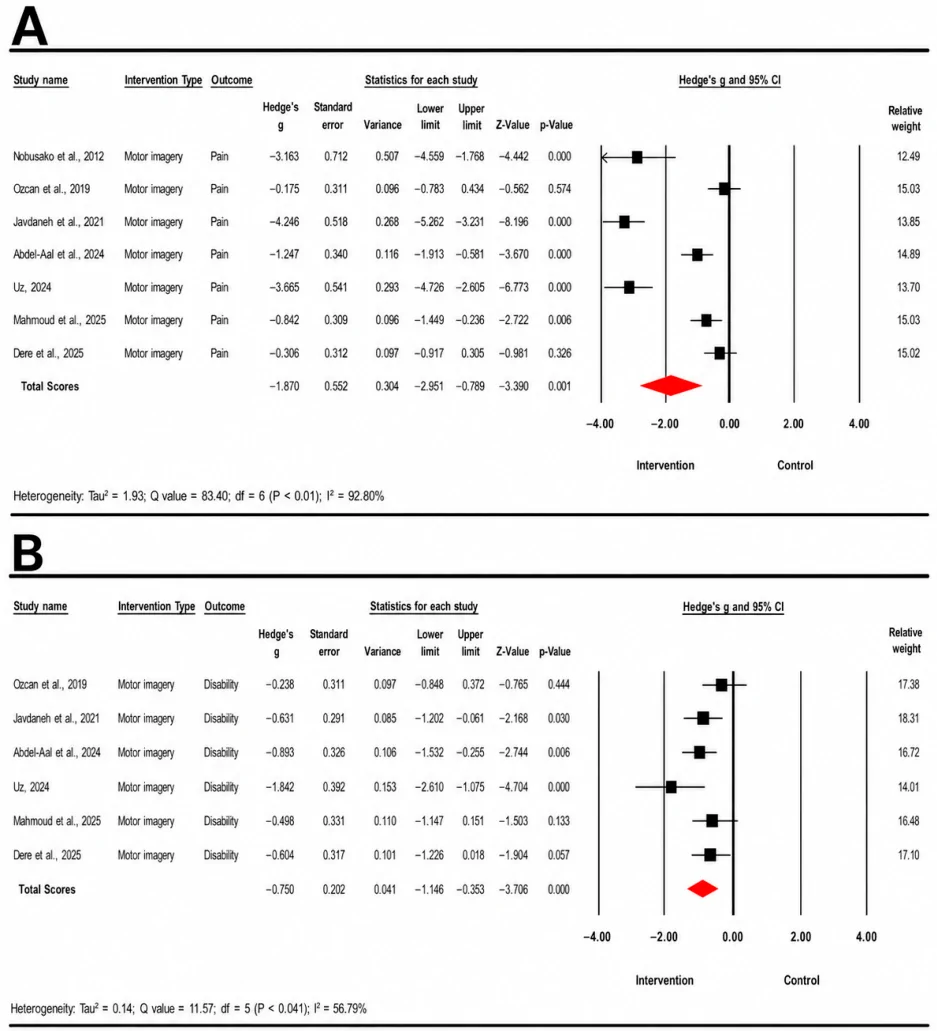
Forest plots showing the effects of motor imagery on pain and disability in patients with non-specific spinal pain. CI, confidence interval. Negative Hedges’ g values favor the intervention group. Squares indicate individual study effects and diamonds indicate pooled effects. Horizontal lines represent 95% confidence intervals. (**A**): Pain [41,44,50,51,54,56,57]; (**B**): Disability [41,50,51,54,56,57].

**Figure 4:**
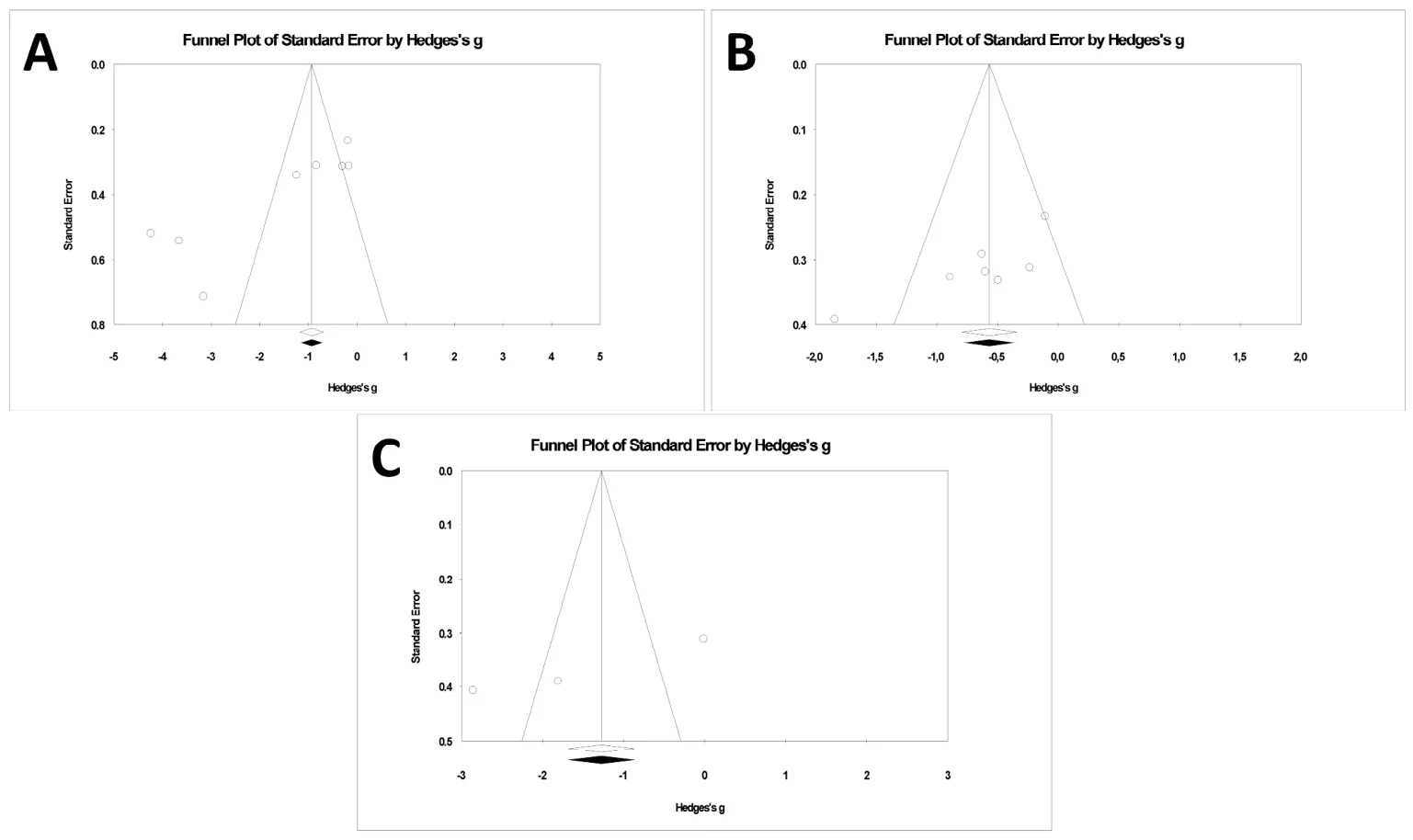
Funnel plots showing the effects of movement representation techniques in patients with non-specific spinal pain. CI, confidence interval. Each circle represents an individual study included in the meta-analysis. The vertical line indicates the pooled effect size, and the diagonal lines represent the pseudo 95% confidence limits. Funnel plot asymmetry may suggest potential publication bias or small-study effects. (**A**): Pain, (**B**): Disability, (**C**): Kinesiophobia.

Eight studies involving 337 participants were included in the meta-analysis for pain intensity. A random-effects model demonstrated a significant overall effect favoring movement representation techniques over control interventions (Hedges’ g = −1.63, 95% confidence interval (CI): −2.56 to −0.70, *p* = 0.001) (Figure 2A). Considerable between-study heterogeneity was observed (Q = 97.15, *p* < 0.01; I^2^ = 92.79%), indicating substantial variability across studies. Given this substantial heterogeneity, an exploratory analysis restricted to motor imagery interventions was subsequently performed for this outcome (Figure 3A; see Section 3.4.4). Publication bias analyses suggested potential funnel plot asymmetry (Figure 4A). Begg and Mazumdar’s rank correlation test demonstrated significant asymmetry (Kendall’s tau = −0.64, *p* = 0.026), and Egger’s regression test was also significant (intercept = −10.07, *p* = 0.003), indicating potential small-study effects. Duval and Tweedie’s trim-and-fill analysis did not identify any missing studies. With only eight trials, however, these procedures have limited power, and the absence of imputed studies should not be interpreted as evidence against small-study effects.

#### 3.4.2. Disability

Seven studies were included in the meta-analysis for disability outcomes. The pooled analysis demonstrated a significant effect favoring movement representation techniques over control interventions (Hedges’ g = −0.64, 95% CI: −1.03 to −0.26, *p* = 0.001) (Figure 2B). Moderate to substantial heterogeneity was identified across studies (Q = 16.67, *p* = 0.01; I^2^ = 64.01%). Publication bias analyses demonstrated possible funnel plot asymmetry (Figure 4B). Begg and Mazumdar’s rank correlation test was not statistically significant in the two-tailed analysis (Kendall’s tau = −0.52, *p* = 0.099), whereas Egger’s regression test indicated potential small-study effects (intercept = −8.58, *p* = 0.020). Duval and Tweedie’s trim-and-fill analysis did not identify any potentially missing studies. Given that only seven trials were pooled, both the asymmetry tests and the trim-and-fill result are exploratory and do not exclude the presence of small-study effects.

#### 3.4.3. Kinesiophobia

Three studies were included in the meta-analysis for kinesiophobia outcomes. The pooled analysis demonstrated a non-significant effect favoring movement representation techniques compared with control interventions (Hedges’ g = −1.55, 95% CI: −3.25 to 0.16, *p* = 0.075) (Figure 2C). Considerable heterogeneity was observed across studies (Q = 33.55, *p* < 0.001), with the calculated I^2^ indicating very high heterogeneity (I^2^ = 94.04%). The funnel plot for kinesiophobia is presented for completeness (Figure 4C). Formal assessment of publication bias was not undertaken for this outcome, as tests for funnel plot asymmetry and the trim-and-fill procedure are uninformative when only three trials are available.

#### 3.4.4. Sensitivity Analysis Restricted to Motor Imagery Interventions

To further investigate whether intervention type contributed to the observed heterogeneity, an exploratory analysis restricted to motor imagery interventions was conducted for pain intensity and disability outcomes. Because only one trial in each model evaluated a non-motor-imagery technique, this constitutes a restriction to the predominant modality rather than a formal comparison between techniques. Subgroup analyses by spinal region, control type, and study design were not feasible, as too few trials contributed to each; the pooled estimates are therefore regarded as exploratory.

For pain intensity, seven studies evaluating motor imagery interventions were included. The pooled analysis demonstrated a statistically significant reduction in pain compared with the control interventions (Hedges’ g = −1.87, 95% CI: −2.95 to −0.79, *p* = 0.001). High between-study heterogeneity was observed (Q = 83.4, *p* < 0.01; I^2^ = 92.80%) (Figure 3A).

For disability, six studies evaluating motor imagery interventions were included. The pooled analysis demonstrated a statistically significant improvement in disability compared with the control interventions (Hedges’ g = −0.75, 95% CI= −1.15 to −0.35, *p* < 0.001). Moderate between-study heterogeneity was observed (Q = 11.57, *p* < 0.04; I^2^ = 56.79%) (Figure 3B).

### 3.5. Risk of Bias Assessment

**Figure 5:**
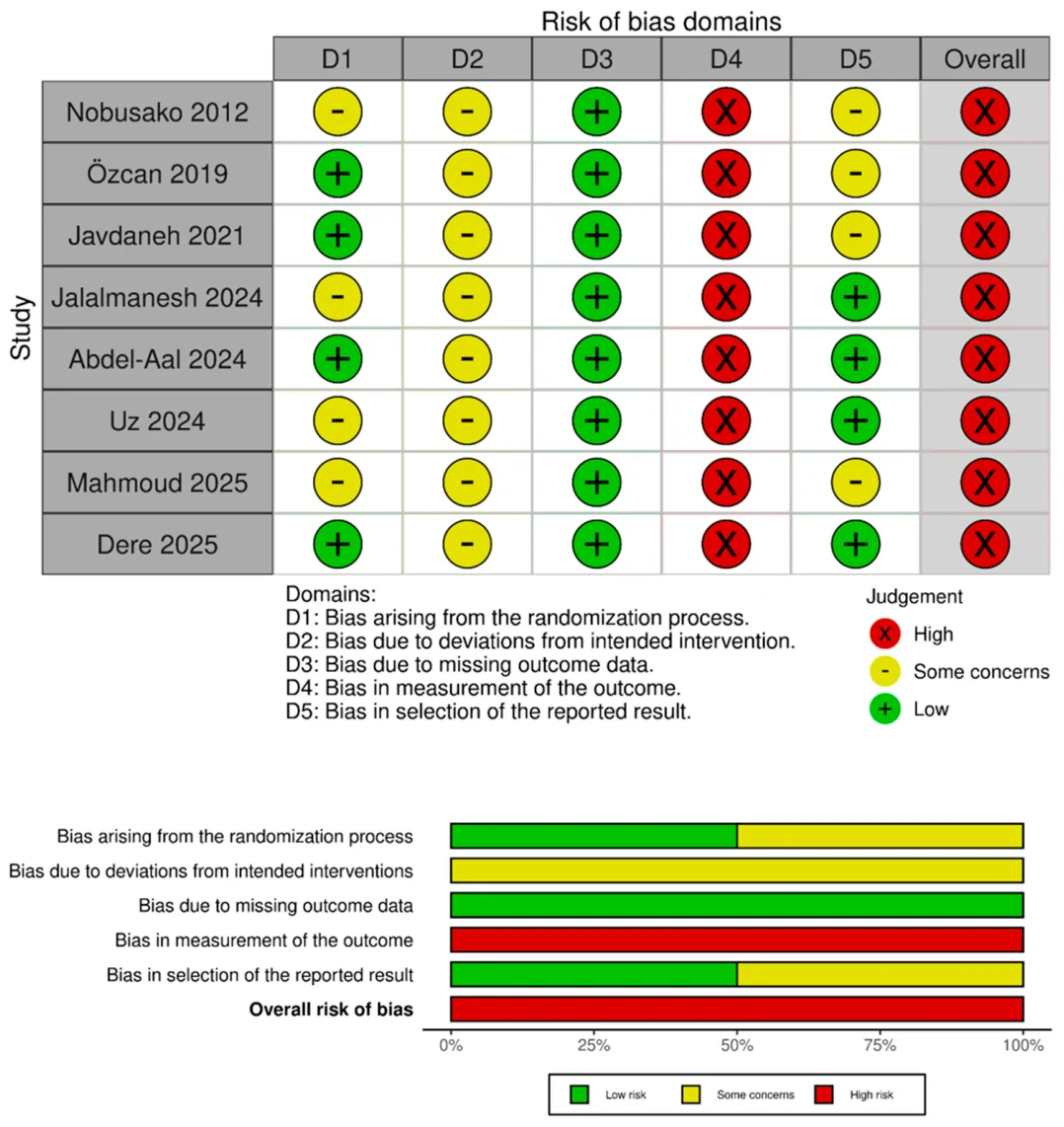
Risk of bias assessment for pain intensity. *Traffic-light plot and weighted summary plot of RoB 2 judgments for pain intensity across included studies* [41,44,50,51,54,55,56,57].

**Figure 6:**
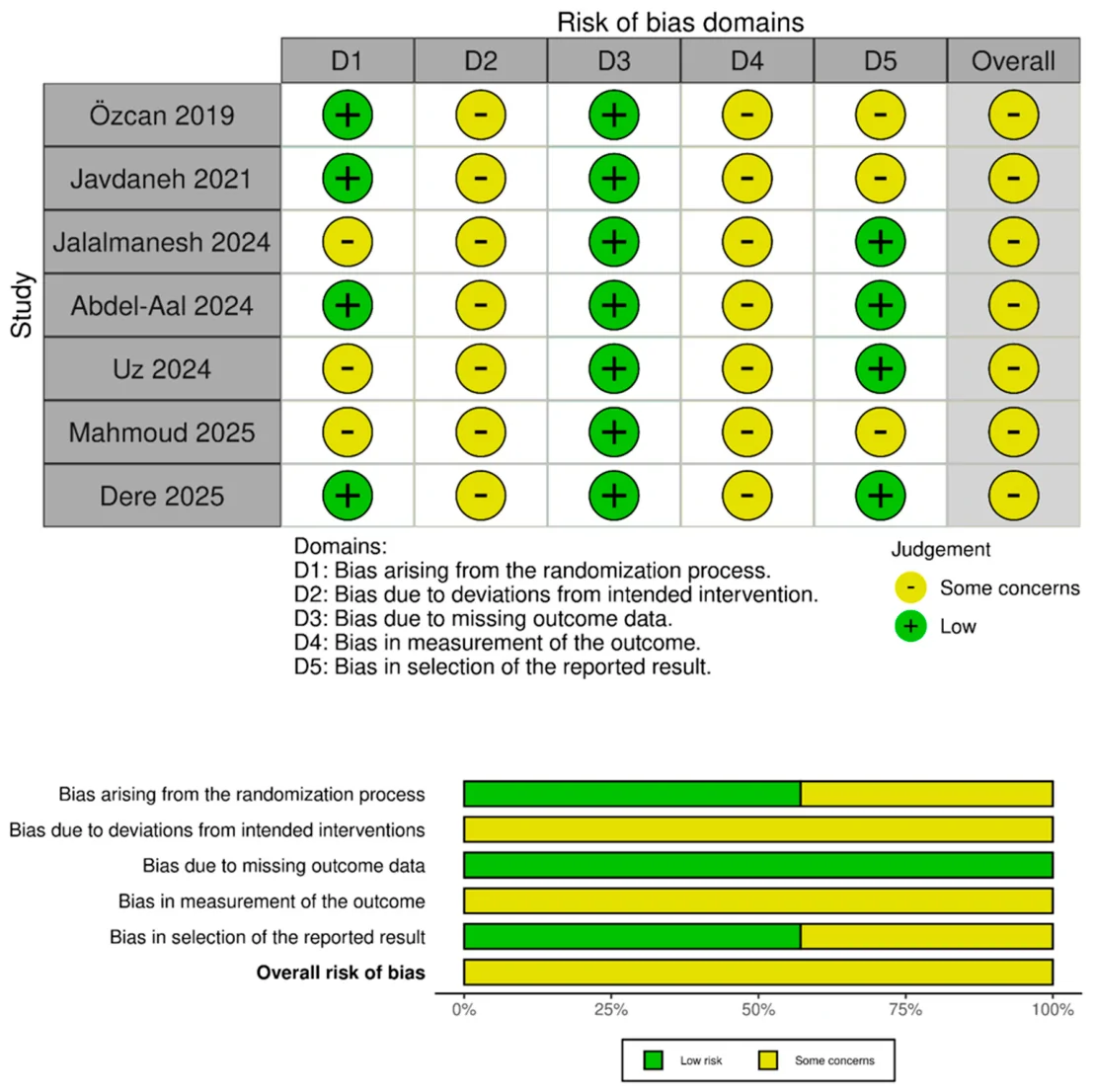
Risk of bias assessment for disability. *Traffic-light plot and weighted summary plot of RoB 2 judgments for disability across included studies* [41,50,51,54,55,56,57].

**Figure 7:**
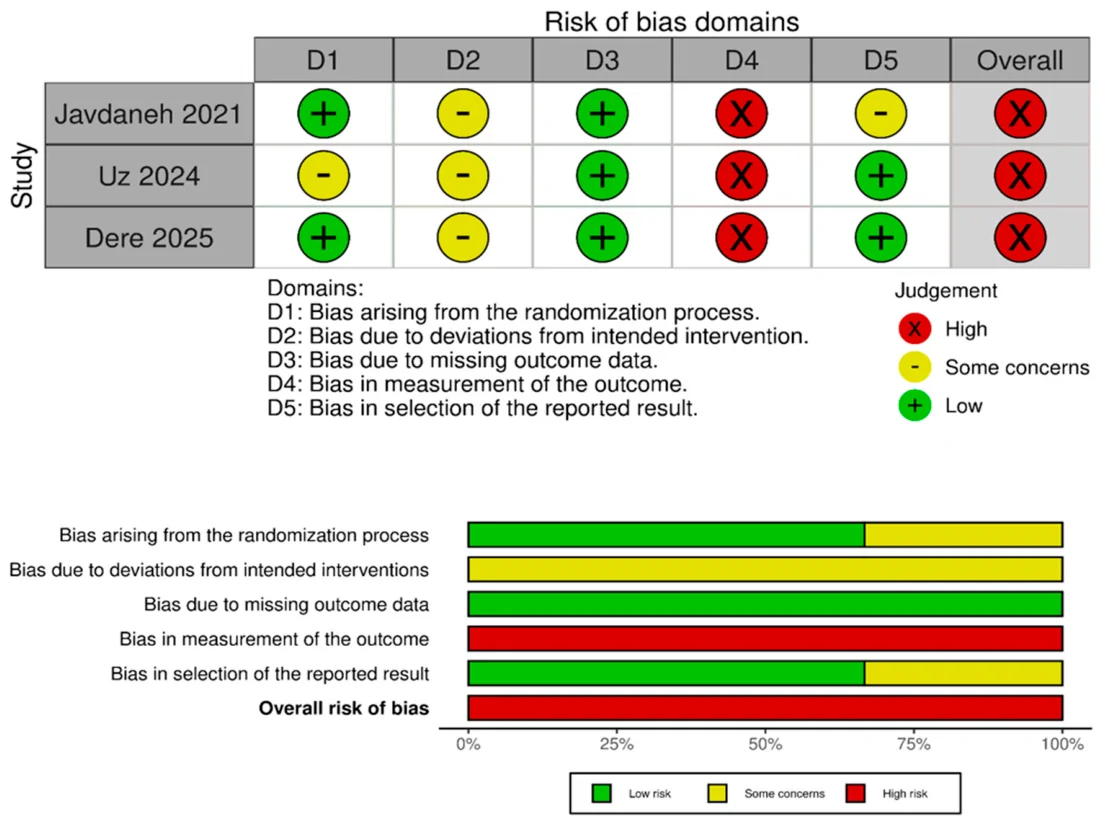
Risk of bias assessment for kinesiophobia. *Traffic-light plot and weighted summary plot of RoB 2 judgments for kinesiophobia across included studies* [41,50,56].

Risk-of-bias judgments were performed separately for pain intensity, disability, and kinesiophobia outcomes. Notably, the study by Daskalaki et al. [58] was excluded from the meta-analysis because it did not use true randomization and was of poor methodological quality; as RoB 2 is validated for randomized trials only, it was not included in the risk-of-bias assessment. Study-level traffic-light plots and weighted summary plots are presented in Figure 5, Figure 6 and Figure 7. For pain intensity (Figure 5), all eight studies were judged as having an overall high risk of bias, primarily due to concerns related to outcome measurement in non-blinded trials using self-reported pain scales. Randomization procedures were generally rated as low risk or with some concerns. For disability outcomes (Figure 6), all included studies were judged as having some concerns overall. The main concerns were related to lack of participant and therapist blinding, incomplete reporting of allocation concealment, and absence of accessible prospective analysis plans. For kinesiophobia (Figure 7), all three studies were judged as having an overall high risk of bias, mainly due to the use of self-reported outcomes in non-blinded interventions. Given the limited number of studies, these findings should be interpreted cautiously. Overall, the risk-of-bias profile differed according to outcome type, with higher risk observed for pain intensity and kinesiophobia outcomes compared with disability outcomes.

### 3.6. Certainty of Evidence

The certainty of evidence was rated as very low for pain intensity, disability, and kinesiophobia (Table 3). For pain intensity, certainty was reduced because of risk of bias, substantial unexplained heterogeneity, indirectness, and suspected publication bias. For disability, the main concerns were risk of bias, heterogeneity, indirectness, and imprecision. For kinesiophobia, certainty was limited by risk of bias, substantial heterogeneity, indirectness, and considerable imprecision. Therefore, confidence in all pooled estimates was very limited.

## 4. Discussion

This systematic review and meta-analysis evaluated the effectiveness of MRTs in individuals with CNSP. The pooled findings demonstrated that MRTs resulted in a large and statistically significant reduction in pain intensity (Hedges’ g = −1.63) and a moderate improvement in disability (Hedges’ g = −0.64) compared with control interventions. In exploratory analyses restricted to motor imagery interventions, significant effects were also observed for pain intensity (Hedges’ g = −1.87; 95% CI: −2.95 to −0.79) and disability (Hedges’ g = −0.75; 95% CI: −1.15 to −0.35), with effect directions consistent with the primary analyses. Although the number of included studies was limited, the methodological quality of the studies ranged from moderate to good. Publication bias analyses suggested possible funnel plot asymmetry and small-study effects for pain intensity and disability; given the small number of trials, however, these analyses cannot exclude selective reporting. Risk-of-bias assessments further indicated a higher risk of bias for pain intensity and kinesiophobia outcomes, mainly due to the use of self-reported measures in non-blinded interventions. The findings also suggest that MRTs, when used as an adjunct to exercise or conventional physiotherapy, may provide beneficial effects on pain intensity and disability; however, the large pooled effect size observed for kinesiophobia did not reach statistical significance, potentially reflecting the limited number of included studies and substantial between-study heterogeneity. Therefore, all findings should be interpreted with caution.

At the individual study level, five of the eight pooled trials reported statistically significant reductions in pain intensity favoring MRTs. In the three trials that did not reach statistical significance, MRT was added to an active exercise or motor control programme, so the estimate reflects its incremental rather than total effect [41,55,57]. The reduction in pain intensity observed in the present meta-analysis is consistent with the umbrella review conducted by Cuenca-Martínez et al. (2022) [59], which similarly demonstrated that movement representation methods were effective in reducing chronic musculoskeletal pain, with a comparable large effect size (SMD = −1.47; 95% CI: −2.05 to −0.88). It should be noted, however, that the findings of the present review are specific to CNSP and cannot be generalized to conditions such as neuropathic pain, phantom limb pain, or post-stroke pain, in which MRT has demonstrated inconsistent results [59]. The substantial heterogeneity observed for pain intensity (I^2^ = 92.79%) may partly reflect the considerable variability in motor imagery session duration and frequency across studies, the absence of standardized assessments of participants’ motor imagery ability prior to intervention, and the inclusion of both cervical and lumbar pain populations within a single pooled analysis. Furthermore, the relatively small sample sizes and high risk of bias across included studies may have contributed to an overestimation of the pooled effect size. The underlying mechanism by which MRT produces hypoalgesia in patients with musculoskeletal pain is thought to involve the reorganization of cortical processes within the primary somatosensory cortex, which is disrupted by chronic pain; specifically, the sensorimotor inputs generated through MRTs are proposed to normalize maladaptive cortical representations, thereby attenuating pain processing at the central level [60,61,62].

The moderate effect size observed for disability (Hedges’ g = −0.64) indicates that MRT positively influences not only pain intensity but also functional capacity. At the individual study level, six of the seven studies reporting disability outcomes demonstrated statistically significant results favoring MRTs. Özcan et al. (2019), however, reported no statistically significant between-group difference on the Neck Disability Index, consistent with the absence of a significant between-group difference in pain intensity in the same trial [57]. The neurophysiological basis underlying the effect of MRTs on disability may be explained by the extensive involvement of the mirror neuron system in motor learning processes through movement representation [63,64]; the cortical–subcortical networks responsible for the planning, execution, and automatisation of real movements have been reported to share similar neurophysiological activity during movement representation [65]. Given that this neurophysiological activity has been shown to be influenced by physical, cognitive-evaluative, and motivational-affective variables, the favorable effects of MRT on muscle strength and its capacity to enhance motor learning processes may be considered additional mechanisms contributing to functional improvement [66,67,68].

Although the large effect size observed for kinesiophobia (Hedges’ g = −1.55) did not reach statistical significance (*p* = 0.075), this is likely attributable to the very limited number of included studies (n = 3) and the substantial between-study heterogeneity (I^2^ = 94.04%). At the individual study level, Javdaneh et al. (2021) [50] and Uz (2024) [56] reported statistically significant reductions in kinesiophobia favoring MRTs (*p* < 0.001 for both), whereas Dere et al. (2025) [41] found no significant between-group difference (*p* = 0.417). Kinesiophobia has been reported to be prevalent across a range of musculoskeletal pain conditions, with higher levels strongly associated with greater pain intensity, higher disability, and lower quality of life [69,70,71,72,73]. Furthermore, a negative correlation between kinesiophobia levels and both kinesthetic and visual motor imagery ability has been documented in patients with CLBP, alongside significantly impaired motor imagery capacity compared with healthy individuals [74]. Similarly, reduced motor imagery ability has been reported in young adults with CNP, with this impairment becoming more pronounced as disability levels increase [75]. On this basis, it may be hypothesized that MRTs, by enhancing the capacity to generate visual and kinesthetic motor images, could exert a favorable effect on kinesiophobia. The non-significant pooled result observed in the present meta-analysis should therefore not be interpreted as an absence of effect, but rather as a reflection of insufficient statistical power arising from the limited number of available studies. Future research incorporating larger sample sizes, long-term follow-up, and standardized kinesiophobia assessment protocols within randomized controlled trials is warranted to draw more definitive conclusions.

The meta-analysis was restricted to pain intensity, disability, and kinesiophobia because these were the only domains reported consistently enough across trials to permit pooling; pain and pain-related disability are also the core outcome domains recommended for chronic pain trials. Disability was operationalized through the Neck Disability Index for cervical and the Oswestry Disability Index for lumbar presentations, which share the same construct and scoring direction and could therefore be combined. Other measures addressed different constructs (SF-36, MIQ-3), captured observed performance (Timed Up and Go), or were reported by too few trials to permit synthesis (spinal range of motion).

From a clinical perspective, the addition of MRTs to conventional physiotherapy may be considered a feasible and potentially beneficial adjunct intervention for individuals with CNSP, particularly in those presenting with features of central sensitization such as widespread pain, heightened pain sensitivity, and impaired sensorimotor integration. However, clinicians should be aware that the current evidence base remains limited, and individualized clinical reasoning is essential when selecting MRT modalities and determining appropriate dosage parameters.

The present systematic review and meta-analysis has several limitations that should be acknowledged when interpreting the findings. First, considerable between-study heterogeneity was observed for pain intensity (I^2^ = 92.79%) and kinesiophobia (I^2^ = 94.04%) outcomes, which may reflect differences in participant characteristics, intervention protocols, and outcome measurement tools across studies. Second, the number of included studies remains limited, particularly for disability (n = 7) and kinesiophobia (n = 3) outcomes, which restricts the statistical power and generalizability of the pooled findings. Third, the scope of the present review was confined to the cervical and lumbar regions, as no eligible studies on thoracic spinal pain were identified; this limits the generalizability of the findings to all spinal regions. Fourth, intervention duration varied substantially across studies, ranging from three to ten weeks. The quantitative syntheses primarily used assessments obtained immediately after completion of treatment. One exception was Nobusako et al. [44], for which an immediate post-treatment group-level summary was not available in an extractable form and the earliest available post-intervention assessment, obtained 15 days after the final treatment session, was used. This difference in assessment timing should be considered when interpreting the pooled pain estimate. Longer-term effects could not be synthesized separately because follow-up data were reported by too few trials. Fifth, blinding of participants and therapists was not feasible in any of the included studies given the nature of the interventions, and the majority of outcome measures relied on self-report tools, which introduces a potential risk of performance and detection bias. Although blinding of outcome assessors represents an important means of minimizing this bias, it was not consistently implemented across studies. Sixth, the control group interventions differed across studies, incorporating varying combinations of electrotherapy, range-of-motion exercises, stretching, mobilization, and strengthening exercises, which may have contributed to between-study heterogeneity and influenced the magnitude of observed between-group differences. Seventh, due to the limited number of eligible studies, all MRT modalities—including motor imagery, mirror therapy, and motor imagery-driven gaze direction recognition—were pooled as a single intervention category in the meta-analysis; however, these techniques are underpinned by distinct neurophysiological mechanisms and may differ in their therapeutic potential. Eighth, the operational definitions of chronicity varied across trials, and this variation in case definition represents an additional source of clinical heterogeneity that could not be addressed analytically given the small number of trials. Ninth, the search was restricted to English- and Turkish-language publications, which may have introduced language bias; relevant trials published in other languages may have been missed. Finally, all eight pooled randomized trials were judged as having a high risk of bias or some concerns in at least one domain of the RoB 2 tool. The single non-randomized trial had poor methodological quality, with a PEDro score of 2/10, and was excluded from all pooled analyses because it used semi-randomized rather than true randomized allocation. Consequently, neither this study nor its poor methodological quality influenced the pooled effect estimates [58]. Notwithstanding these limitations, grey literature sources including ProQuest Dissertations & Theses and the Turkish Council of Higher Education Thesis Centre were searched in addition to database searches to minimize publication bias.

Future research should address these limitations by conducting larger, adequately powered, and rigorously designed randomized controlled trials incorporating standardized MRT protocols, active control conditions, and long-term follow-up assessments. Studies directly comparing different MRT modalities in individuals with CNSP, and extending coverage to the thoracic region, are particularly warranted. Furthermore, the incorporation of neuroimaging techniques to elucidate the neural correlates of MRTs and the investigation of optimal dosage and frequency parameters would substantially advance the mechanistic understanding of these interventions.

## 5. Conclusions

In conclusion, this systematic review and meta-analysis provides preliminary evidence that movement representation techniques, when used as an adjunct to exercise or conventional physiotherapy, may reduce pain intensity and improve disability in individuals with CNSP. However, the GRADE assessment rated the certainty of evidence as very low for pain intensity, disability, and kinesiophobia. Given the high risk of bias, substantial heterogeneity, and limited number of included studies, these findings should be considered exploratory and interpreted with caution. Further methodologically rigorous randomized controlled trials with larger samples, standardized protocols, and long-term follow-up are needed before definitive clinical recommendations can be made.

## Figures and Tables

**Table 1:** PEDro scale for included studies.

Studies	Q1	Q2	Q3	Q4	Q5	Q6	Q7	Q8	Q9	Q10	Q11	PEDro Score
Nobusako et al., 2012 [44]	Y	Y	N	Y	N	N	Y	Y	Y	Y	Y	7/10
Özcan et al., 2019 [57]	Y	Y	Y	N	N	N	Y	Y	N	Y	Y	6/10
Javdaneh et al., 2021 [50]	Y	Y	Y	Y	N	N	Y	Y	N	Y	Y	7/10
Jalalmanesh et al., 2024 [55]	Y	Y	Y	N	N	N	N	Y	Y	Y	Y	6/10
Daskalaki et al., 2024 [58]	N	N	N	N	N	N	N	N	N	Y	Y	2/10
Abdel-Aal et al., 2024 [51]	Y	Y	Y	Y	N	N	Y	Y	Y	Y	Y	8/10
Uz, 2024 [56]	Y	Y	N	Y	N	N	Y	Y	N	Y	Y	6/10
Mahmoud et al., 2025 [54]	Y	Y	Y	Y	Y	N	Y	Y	N	Y	Y	8/10
Dere et al., 2025 [41]	Y	Y	Y	Y	N	N	Y	Y	N	Y	Y	7/10

**Table 2:** Characteristics of included studies.

Author and Year	Population -Sample Size and Sex -Disease -Mean Age	-Intervention Groups (n) -Study Design	Time Points for Assessments	Outcome Measures	Results Control (1) and Intervention (2), Respectively Between Group *p* Values
Nobusako et al., 2012 [44]	- N = 17 (9 male and 8 female) - Chronic neck pain, defined as neck symptoms lasting >6 months - Mean age = 54.4 ± 11.6 (control); 50.3 ± 17.5 (intervention group)	- (1) Control group (Physical therapy only; *n* = 8) - (2) Intervention group (a motor imagery-driven gaze direction recognition task; *n* = 9) - A pilot randomized controlled study	- Baseline - Assessments before and after each session (11 sessions over 3 weeks) - Final assessment (15-day follow-up)	- VAS pain during right neck rotation (0–100) - VAS pain during left neck rotation (0–100) - Active range of motion during right neck rotation - Active range of motion during left neck rotation	*A Motor Imagery-Driven Gaze Direction Recognition Task > Control* - VAS Pain During Right Neck Rotation (Pre-test and Final assessment [15-day follow-up] mean score ± standard deviation) (1) 51.1 ± 25.2 and 41.6 ± 22.0; (2) 66.1 ± 14.5 and 6.6 ± 8.5; *p* < 0.001 (ANOVA, F_(3.937, 59.062)_ = 8.374) - VAS Pain During Left Neck Rotation (Pre-test and Final assessment [15-day follow-up] mean score ± standard deviation) (1) 53.9 ± 15.3 and 46.5 ± 10.1; (2) 48.1 ± 19.9 and 6.6 ± 8.6; *p* = 0.012 (ANOVA, F_(3.757, 56.356)_ = 3.614) - Active Range Of Motion During Right Neck Rotation (Pre-test and Final assessment [15-day follow-up] mean score ± standard deviation) (1) 46.4 ± 7.9 and 47.5 ± 7.6; (2) 38.3 ± 8.7 and 62.5 ± 4.6; *p* < 0.001 (ANOVA, F_(4.797, 71.952)_ = 11.051) - Active Range Of Motion During Right–Left Rotation (Pre-test and Final assessment [15-day follow-up] -mean score ± standard deviation) (1) 44.9 ± 6.8 and 45.8 ± 5.7; (2) 46.4 ± 6.7 and 62.4 ± 4.5; *p* < 0.001 (ANOVA, F_(3.616, 54.237)_ = 6.626)
Özcan et al., 2019 [57]	- N = 40 (7 male and 33 female) - Chronic non-specific neck pain, defined as symptoms lasting ≥3 months - Mean age = 19.70 ± 1.17 (exercise group) - Mean age = 20.10 ± 1.33 (exercise + MI group)	- (1) Exercise group (n = 20) - (2) Exercise+ Motor Imagery Training group (n = 20) - Randomized single-blind controlled trial	- Baseline - Final assessment (4 weeks, 5 days/week)	- VAS (Pain, 0–10) - Neck Disability Index - Motor Imagery Questionnaire-3 (MIQ-3) - SF-36 quality of life	*Exercise group ~ =* Exercise+ Motor Imagery Training - Visual Analog Scale [Pre-test and Final assessment—median (minimum-maximum)] (1) 4.35 (0.9–7.2) and 1.0 (0.0–4.5); (2) 4.55 (1.8–7.7) and 1.5 (0.0–5.9); *p* = 0.369, Not statistically significant - Neck Disability Index (Pre-test and Final assessment -mean score ± standard deviation) (1) 20.50 ± 7.72 and 7.98 ± 5.51; (2) 24.20 ± 9.56 and 10.40 ± 5.02; *p* = 0.608, Not statistically significant - Motor imagery ability (Pre-test and Final assessment -mean score ± standard deviation) ○ Internal visual imagery: (1) 5.20 ± 0.79 and 5.70 ± 0.69; (2) 5.80 ± 0.76 and 6.20 ± 0.62; *p* = 0.541, Not statistically significant ○ Kinesthetic visual imagery: (1) 5.30 ± 0.98 and 5.70 ± 0.77; (2) 5.80 ± 0.77 and 6.20 ± 0.69; *p* = 0.862, Not statistically significant - SF-36 quality of life (Pre-test and Final assessment -mean score ± standard deviation) ○ SF-36 Pain: (1) 63.50 ± 15.89 and 80.80 ± 9.30; (2) 59.10 ± 16.56 and 71.90 ± 13.35; *p* = 0.401, Not statistically significant ○ SF-36 General Health: (1) 63.70 ± 18.10 and 72.85 ± 13.49; (2) 54.50 ± 20.24 and 65.45 ± 16.29; *p* = 0.612, Not statistically significant ○ SF-36 Physical Function: (1) 88.25 ± 9.64 and 92.50 ± 7.52; (2) 86.50 ± 10.89 and 92.75 ± 8.81; *p* = 0.924, Not statistically significant
Javdaneh et al., 2021 [50]	- N = 72 (36 male and 36 female) - Chronic non-specific neck pain, defined as ongoing bilateral neck pain lasting >3 months (VAS 30–70 mm) - Mean age = 33.41 ± 6.77 (control group) - Mean age = 34.58 ± 5.37 (exercise group) - Mean age = 32.25 ± 8.12 (exercise + motor imagery training group)	- (1) Control (no intervention; n = 24) - (2) Neck stabilization exercises (n = 24) - (3) Neck stabilization exercises+ motor imagery training (n = 24) - Randomized controlled study	- Baseline - Final assessment (6 weeks, 3 days/week)	- VAS (Pain, 0–100 mm) - Neck Disability Index - Tampa Scale of Kinesiophobia	*Neck stabilization exercises+ motor imagery training>* Neck stabilization exercises *> Control* - Visual Analog Scale (Pre-test and Final assessment -mean score ± standard deviation) (1) 61.40 ± 6.04 and 59.95 ± 6.17; (2) 60.15 ± 6.30 and 32.55 ± 5.65; (3) 62.60 ± 6.15 and 14.45 ± 3.66; *p* < 0.001 - Neck Disability Index (Pre-test and Final assessment -mean score ± standard deviation) (1) 25.75 ± 2.71 and 26.20 ± 2.12; (2) 27.80 ± 2.55 and 16.10 ± 2.94; (3) 26.12 ± 2.42 and 11.10 ± 2.98; *p* < 0.001 - Tampa Scale of Kinesiophobia (Pre-test and Final assessment -mean score ± standard deviation) (1) 53.60 ± 5.23 and 53.75 ± 4.51; (2) 50.65 ± 5.55 and 34.65 ± 3.37; (3) 52.15 ± 4.40 and 25.60 ± 3.87; *p* < 0.001
Jalalmanesh et al., 2024 [55]	- N = 72 (22 male and 50 female) - Chronic non-specific low back pain, defined as symptoms lasting ≥3 months (NRS ≥ 3) - Mean age = 51.00 ± 8.5 (control group) - Mean age = 50.00 ± 8.9 (mirror therapy group)	- (1) Control group (movement correction exercises without mirror (n = 36) - (2) Movement correction exercises with mirror therapy (n = 36) - Randomized controlled study	- Baseline - Final assessment (post-intervention; 10 weeks, 3 days/week) - 3-month follow-up	- Numerical rating scale (0–10) - Oswestry Disability Index (0–100%) - Chronic Pain Questionnaire	*Movement correction exercises with mirror therapy > Control* - Numerical rating scale (Pre-test and Final assessment -mean score ± standard deviation) (1) 7.9 ± 1.7 and 3.8 ± 2.7; (2) 7.8 ± 1.9 and 4.2 ± 2.4; *p* = 0.003 - Oswestry Disability Index (Pre-test and Final assessment -mean score ± standard deviation) (1) 13.00 ± 5.1 and 6.1 ± 5.0; (2) 16.3 ± 4.8 and 8.7 ± 7.4; *p* < 0.001 - Chronic Pain Questionnaire (Pre-test and Final assessment -mean score ± standard deviation) (1) 89.17 ± 12.76 and 52.05 ± 8.64; (2) 92.42 ± 12.01 and 42.23 ± 9.79; *p* = 0.003
Daskalaki et al., 2024 [58]	- N = 30 (30 female) - Chronic low back pain, defined as persistent pain during the previous 3 months - Mean age = 46.00 ± 7.67 (control group) Mean age = 46.50 ± 7.96 (exercise group) Mean age = 50.75 ± 8.40 (exercise +mental imagery group)	- (1) Control group (no intervention, n = 10) - (2) Exercise group (n = 10) - (3) Exercise + mental imagery group (n = 10) - Semi-randomized controlled trial	- Baseline - Final assessment-11 weeks (18 sessions/9 weeks + 2-week follow-up)	- Numerical rating scale (0–10) - Functional Performance Test - SF-36 Quality of Life	*Exercise+ mental imagery training > Exercise > Control* - Numerical rating scale (Pre-test and Final assessment -mean score ± standard deviation) (1) 3.60 ± 1.77 and 4.00 ± 2.05; (2) 3.50 ± 2.99 and 2.70 ± 2.16; (3) 5.20 ± 1.40 and 2.80 ± 1.75; *p* = 0.022 (ANOVA, F_(6–81)_ = 2.633) - Functional Performance Test [Times Up and Go Test (Pre-test and Final assessment -mean score ± standard deviation)] (1) 6.32 ± 0.6 and 6.30 ± 0.74; (2) 5.87 ± 1.42 and 5.26 ± 1.08; 3) 6.23 ± 0.63 and 5.51 ± 0.66; *p* = 0.003 (ANOVA, F_(6–81)_ = 3.690) - SF-36 quality of life (Pre-test and Final assessment -mean score ± standard deviation) ○ SF-36 Pain: (1) 60.75 ± 21.21 and 56.50 ± 19.87; (2) 63.00 ± 15.22 and 76.75 ± 14.67; (3) 56.75 ± 17.87 and 67.00 ± 19.14; *p* = 0.010 (ANOVA, F_(6–81)_ = 3.053) SF-36 General Health: (1) 69.00 ± 12.43 and 69.50 ± 15.17; (2) 70.00 ± 21.34 and 76.50 ± 13.34; (3) 69.00 ± 12.86 and 69.00 ± 15.23; *p* > 0.005 SF-36 Physical Function: (1) 80.00 ± 8.50 and 79.00 ± 13.08; (2) 70.50 ± 15.71 and 83.00 ± 10.05; (3) 74.00 ± 18.23 and 88.00 ± 10.05; *p* < 0.004 (ANOVA, F_(6–81)_ = 3.442)
Abdel-Aal et al., 2024 [51]	- N = 60 (25 male and 35 Female) - Chronic neck pain, defined as persistent cervical pain lasting >3 months - Mean age = 33.25 ± 6.63 (control group) - Mean age = 33.95 ± 7.8 (eye cervical re-education exercises group) - Mean age = 33.35 ± 7.21 (motor imagery group)	(1) Control group (n = 20) (2) Eye cervical re-education exercises group (n = 20) (3) Motor imagery group (n = 20) - Randomized controlled study	- Baseline - Final assessment -four weeks	- VAS (Pain, 0–100 mm) - Neck Disability Index	Eye cervical re-education exercises group > Motor imagery group > Control - Visual Analog Scale (Pre-test and Final assessment -mean score ± standard deviation) (1) 71.80 ± 8.02 and 53.25 ± 9.3; (2) 79.9 ± 7.41 and 27.65 ± 5.91; (3) 74.0 ± 4.54 and 44.9 ± 7.14, *p* = 0.001 (ANOVA, F_(2–57)_ = 59.35) - Neck Disability Index (Pre-test and Final assessment t-mean score ± standard deviation) (1) 37.7 ± 3.74 and 22.3 ± 3.89; (2) 35.3 ± 4.14 and 9.9 ± 3.13; (3) 37.25 ± 4.17 and 18.05 ± 4.43, *p* = 0.001 (ANOVA, F(2–57) = 53.45) - Range of Motion (Pre-test and Final assessment -mean score ± standard deviation) ○ Flexion: (1) 33.5 ± 4.94 and 39.5 ± 5.5; (2) 32.3 ± 3.51 and 47.6 ± 3.93; (3) 34.8 ± 2.78 and 43.4 ± 3.5; *p* = 0.001 (ANOVA, F_(2–57)_ = 17) Extension: (1) 32.0 ± 5.35 and 37.7 ± 5.03; (2) 34.5 ± 6.8 and 53.70 ± 5.52; (3) 31.9 ± 6.34 and 44.70 ± 6.88; *p* = 0.001 (ANOVA, F_(2–27)_ = 37.6) Right Lateral Flexion: (1) 29.5 ± 5.23 and 33.5 ± 4.81; (2) 26.8 ± 4.87 and 43.30 ± 4.8; (3) 26.0 ± 4.94 and 37.50 ± 2.59; *p* = 0.001 (ANOVA, F_(2–57)_ = 32.94) Left Lateral Flexion: (1) 30.8 ± 5.96 and 35.0 ± 4.66; (2) 28.35 ± 4.6 and 43. 5 ± 2.89; (3) 27.5 ± 5.02 and 39.3 ± 4.12; *p* = 0.001 (ANOVA, F_(2–57)_ = 23.05) Right Rotation: (1) 38.3 ± 5.04 and 43.50 ± 4.63; (2) 38.0 ± 5.84 and 57.3 ± 6.23; (3) 36.0 ± 6.46 and 48.8 ± 5.67; *p* = 0.001 (ANOVA, F_(2–57)_ = 23.05) Left Rotation: (1) 38.4 ± 5.49 and 44.10 ± 4.92; (2) 39.3 ± 5.32 and 59.10 ± 4.7; (3) 37.1 ± 6.27 and 50.45 ± 6.33; *p* = 0.001 (ANOVA, F_(2–57)_ = 39.39)
Uz, 2024 [56]	- N = 36 (17 male and 19 Female) - Chronic non-specific low back pain, described as pain lasting >12 weeks - Mean age = 39.7 ± 8.3 (Exercise group receiving telerehabilitation) - Mean age = 42.6 ± 10.2 (Exercise + motor imagery group receiving telerehabilitation)	(1) Exercise group receiving telerehabilitation (n = 18) (2) Exercise + motor imagery group receiving telerehabilitation (n = 18) - Single blind randomized controlled study	- Baseline - Final assessment -ten weeks	- Visual Analog Scale (0–10) - Oswestry Disability Index (0–100%)	*Exercise + motor imagery > Exercise group* - Visual Analog Scale-Activity (Pre-test and Final assessment -mean score ± standard deviation) (1) 3.44 ± 1.29 and 3.56 ± 0.92; (2) 5.06 ± 1.83 and 2.00 ± 0.77; *p* < 0.001 - Oswestry Disability Index (Pre-test and Final assessment -mean score ± standard deviation) (1) 33.17 ± 17.92 and 34.17 ± 16.71; (2) 37.67 ± 16.02 and 13.56 ± 8.72; *p* < 0.001 - Tampa Scale of Kinesiophobia (Pre-test and Final assessment Post-mean score ± standard deviation) (1) 41.61 ± 4.78 and 42.17 ± 5.00; (2) 38.44 ± 7.56 and 28.00 ± 6.75; *p* < 0.001 (ANOVA, F = 51.241) - Range of Motion (Pre-test and Final assessment-mean score ± standard deviation) ○ Flexion: (1) 66.39 ± 5.09 and 66.94 ± 4.89; (2) 64.17 ± 4.29 and 79.72 ± 1.18; *p* < 0.001 (ANOVA, F =66.407) ○ Extension: (1) 13.33 ± 4.20 and 13.33 ± 3.43; (2) 11.67 ± 2.43 and 23.06 ± 2.51; *p* < 0.001 (ANOVA, F = 62.372) ○ Right Lateral Flexion: (1) 15.56 ± 4.50 and 17.50 ± 3.93; (2) 16.67 ± 2.43 and 29.44 ± 3.38;; *p* < 0.001 (ANOVA, F = 68.868) ○ Left Lateral Flexion: (1) 15.83 ± 4.29 and 17.50 ± 3.93; (2) 16.67 ± 2.43 and 29.72 ± 2.70; *p* < 0.001 (ANOVA, F = 84.284) ○ Right Rotation: (1) 16.94 ± 5.18 and 18.61 ± 5.37; (2) 18.33 ± 3.83 and 37.22 ± 4.61; *p* < 0.001 (ANOVA, F = 85.954) ○ Left Rotation: (1) 17.78 ± 6.69 and 18.89 ± 5.02; (2)18.61 ± 3.35 and 37.22 ± 5.21; *p* < 0.001 (ANOVA, F = 80.412)
Mahmoud et al., 2025 [54]	-N = 44 (23 male and 21 Female) - Chronic mechanical neck pain, defined as symptoms lasting ≥6 months - Mean age = 43.54 ± 2.97 (control group, conventional physical therapy) - Mean age = 41.23 ± 5.28 (Kinesthetic and visual motor imagery group)	(1) Control group (n = 22) (2) Kinesthetic and visual motor imagery group (n = 22) - Randomized controlled study	- Baseline - Final assessment -four weeks	- VAS (Pain, 0–10) - Neck Disability Index	*Motor Imagery > Control* - Visual Analog Scale-Activity (Pre-test and Final assessment -mean score ± standard deviation) (1) 7.59 ± 1.37 and 4.77 ± 0.97; (2) 7.64 ± 1.22 and 3.68 ± 1.61; *p* = 0.01 - Neck Disability Index (Pre-test and Final assessment -mean score ± standard deviation) (1) 19.95 ± 2.77 and 11.55 ± 2.24; (2) 19.5 ± 4.52 and 9.5 ± 3.84; *p* = 0.037
Dere et al., 2025 [41]	- N = 40 (40 Female) - Chronic neck pain, defined as symptoms persisting for ≥3 months with moderate pain at rest (VAS 3–7/10) - Mean age = 55.00 ± 11.64 (control group, motor control exercises) - Mean age = 51.50 ± 9.31 (Motor control exercises + motor imagery group)	(1) Motor control exercises (n = 20) (2) Motor control exercises + motor imagery group (n = 20) - Single blind randomized controlled study	- Baseline - Final assessment (post-treatment; 8 weeks)) - 12-week follow-up	- VAS (Pain, 0–10) - Neck Disability Index	*Motor Imagery > Control* - Visual Analog Scale-Activity (Pre-test and Final assessment -mean score ± standard deviation) (1) 6.51 ± 1.78 and 3.67 ± 1.76; (2) 7.64 ± 1.76 and 4.19 ± 2.13; *p* = 0.01 - Neck Disability Index (Pre-test and Final assessment -mean score ± standard deviation) (1) 15.25 ± 6.67 and 8.25 ± 5.16; (2) 18.30 ± 6.17 and 8.50 ± 3.83; *p* = 0.041 - Tampa Scale of Kinesiophobia (Pre-test and Final assessment -mean score ± standard deviation) (1) 42.55 ± 10.17 and 34.25 ± 10.57; (2) 39.95 ± 5.74 and 31.80 ± 8.18; *p* = 0.417

**Table 3:** GRADE summary of findings.

Outcome	Studies and Participants	Pooled Effect Estimate	Certainty of Evidence
Pain intensity	8 studies; n = 337	Hedges’ g = −1.63 (95% CI: −2.56 to −0.70)	⨁◯◯◯ Very low ᵃ
Disability	7 studies; n = 320	Hedges’ g = −0.64 (95% CI: −1.03 to −0.26)	⨁◯◯◯ Very low ᵇ
Kinesiophobia	3 studies; *n* = 124	Hedges’ g = −1.55 (95% CI: −3.25 to 0.16)	⨁◯◯◯ Very low ᶜ

Abbreviations: CI, confidence interval; GRADE, Grading of Recommendations Assessment, Development and Evaluation. Negative Hedges’ g values favor movement representation techniques. Certainty of evidence is expressed using GRADE symbols, where ⨁◯◯◯ denotes very low certainty (⨁⨁⨁⨁ high, ⨁⨁⨁◯ moderate, ⨁⨁◯◯ low, ⨁◯◯◯ very low) ᵃ Downgraded for very serious risk of bias and inconsistency, serious indirectness, and suspected publication bias. ᵇ Downgraded for serious risk of bias, inconsistency, indirectness, and imprecision. ᶜ Downgraded for very serious risk of bias, inconsistency, and imprecision, and for serious indirectness.

## Data Availability

The data supporting the findings of this study were extracted from the included published articles and thesis records. All data generated or analyzed during this review are included in this article and its Supplementary Materials. Further inquiries can be directed to the corresponding author.

## Supplementary Materials

The following supporting information can be downloaded at: https://www.mdpi.com/article/10.3390/medicina62081534/s1, Table S1: PRISMA 2020 Checklist. Table S2: Full search strategies used for each database and grey literature source. Reference [36] is cited in Supplementary Materials.

## Abbreviations

The following abbreviations are used in this manuscript:

CI	Confidence Interval
CINAHL	Cumulative Index to Nursing and Allied Health Literature
CLBP	Chronic Low Back Pain
CMA	Comprehensive Meta-Analysis
CNP	Chronic Neck Pain
CNSP	Chronic Non-Specific Spinal Pain
GRADE	Grading of Recommendations Assessment, Development and Evaluation
MeSH	Medical Subject Headings
MI	Motor Imagery
MIQ-3	Motor Imagery Questionnaire-3
MRTs	Movement Representation Techniques
NDI	Neck Disability Index
NRS	Numerical Rating Scale
ODI	Oswestry Disability Index
PEDro	Physiotherapy Evidence Database
PICOS	Participants, Interventions, Comparisons, Outcomes, and Study Design
PRESS	Peer Review of Electronic Search Strategies
PRISMA	Preferred Reporting Items for Systematic Reviews and Meta-Analyses
PROSPERO	International Prospective Register of Systematic Reviews
SF-36	Short Form-36 Health Survey
RoB 2	Risk of Bias 2
SMD	Standardized Mean Difference
TSK	Tampa Scale for Kinesiophobia
VAS	Visual Analogue Scale
