# Supplementary material for: Effectiveness of Movement Representation Techniques in Chronic Non-Specific Spinal Pain: A Systematic Review and Meta-Analysis
Source: Medicina (Kaunas). 2026 Aug 9;62(8):1534. doi: 10.3390/medicina62081534 (PMC13515511; doi:10.3390/medicina62081534)
Supplement: Supplementary file 1 [file medicina-62-01534-s001.zip › medicina-4415649 Table S2_Full Search Strategies Used for Each Database and Grey Literature Source .pdf]

Search Strategy (02.03.2026)

**MEDLINE via PubMed**	93
**EBSCO – CINAHL Complete**	115
**SCOPUS**	171
**Cochrane Library**	101
**PEDro**	16
Gray Literature	22
**Total**	**518**
**Duplicates**	180
**Unique**	**338**

PubMed

Search	Query	Results
**#5**	**Search: #3 AND #4**	**93**
**#4**	**Search: "action observation"[tw] OR "explicit motor imagery"[tw] OR "graded motor imagery"[tw] OR "guided imagery"[tw] OR "imagined movements"[tw] OR "implicit motor imagery"[tw] OR "laterality recognition"[tw] OR "laterality training"[tw] OR "mental imagery"[tw] OR "mental practice"[tw] OR "mirror box"[tw] OR "mirror therapy"[tw] OR "mirror training"[tw] OR "mirror visual feedback"[tw] OR "motor imagery"[tw] OR "observational learning"[tw] OR "bodily illusion"[tw] OR "eidetic imagery"[tw] OR "guided imagery"[tw] OR "kinesthetic imagery"[tw] OR "mental imagery"[tw] OR "mental movements"[tw] OR "mental practice"[tw] OR "mental rehearsal"[tw] OR "mental simulation"[tw] OR "mental training"[tw] OR "mirror therap*"[tw] OR "mirror therapy"[tw] OR "motor imagery"[tw] OR "motor observation"[tw] OR "movement observation"[tw] OR "observation training"[tw] OR "perceptual distortion"[tw] OR "visual illusion"[tw] OR "visual imagery"[tw]**	**16,349**
**#3**	**Search: #1 OR #2**	**117,855**
**#2**	**Search: "back ache"[tw] OR "back dysfunction"[tw] OR "back pain"[tw] OR "back strain"[tw] OR "back syndrome"[tw] OR "backache"[tw] OR "cervical pain"[tw] OR "cervical radiculopathy"[tw] OR "cervical spondylopathy"[tw] OR "cervical spondylosis"[tw] OR "cervicalgia"[tw] OR "cervicalgia "[tw] OR "cervicodynia"[tw] OR "chronic back pain"[tw] OR "chronic low back pain"[tw] OR "chronic neck pain"[tw] OR "discogenic pain"[tw] OR "dorsalgia"[tw] OR "low back pain"[tw] OR "lower back pain"[tw] OR "lumbago"[tw] OR "lumbal pain"[tw] OR "lumbalgia"[tw] OR "lumbar pain"[tw] OR "lumbar spine syndrome"[tw] OR "lumbar spondylosis"[tw] OR "lumbodynia"[tw] OR "lumbosacral pain"[tw] OR "lumbosacral syndrome"[tw] OR "mechanical low back pain"[tw] OR "mechanical neck pain"[tw] OR "neck ache"[tw] OR "neck pain"[tw] OR "neck sprain"[tw] OR "neck symptoms"[tw] OR "neckache"[tw] OR "neckpain"[tw] OR "non specific neck pain"[tw] OR "non-specific neck pain"[tw] OR "postural low back pain"[tw] OR "sacral pain"[tw] OR "spinal pain"[tw] OR "spine pain"[tw] OR "tension neck syndrome"[tw] OR "thoracic back pain"[tw] OR "thoracic pain"[tw] OR "thoracolumbar pain"[tw] OR "trapezius myalgia"[tw] OR "upper back pain"[tw] OR "vertebral pain"[tw] OR "whiplash"[tw]**	**117,609**
**#1**	**Search: "neck pain"[mesh] or "back pain"[mesh] or "low back pain"[mesh] or "whiplash injuries"[mesh]**	**60,064**

EBSCO - CINAHL Complete

Search	Query	Results
**S1**	(("back ache") OR ("back dysfunction") OR ("back pain") OR ("back strain") OR ("back syndrome") OR ("backache") OR ("cervical pain") OR ("cervical radiculopathy") OR ("cervical spondylopathy") OR ("cervical spondylosis") OR ("cervicalgia") OR ("cervicalgia ") OR ("cervicodynia") OR ("chronic back pain") OR ("chronic low back pain") OR ("chronic neck pain") OR ("discogenic pain") OR ("dorsalgia") OR ("low back pain") OR ("lower back pain") OR ("lumbago") OR ("lumbal pain") OR ("lumbalgia") OR ("lumbar pain") OR ("lumbar spine syndrome") OR ("lumbar spondylosis") OR ("lumbodynia") OR ("lumbosacral pain") OR ("lumbosacral syndrome") OR ("mechanical low back pain") OR ("mechanical neck pain") OR ("neck ache") OR ("neck pain") OR ("neck sprain") OR ("neck symptoms") OR ("neckache") OR ("neckpain") OR ("non specific neck pain") OR ("non-specific neck pain") OR ("postural low back pain") OR ("sacral pain") OR ("spinal pain") OR ("spine pain") OR ("tension neck syndrome") OR ("thoracic back pain") OR ("thoracic pain") OR ("thoracolumbar pain") OR ("trapezius myalgia") OR ("upper back pain") OR ("vertebral pain") OR ("whiplash")) AND (("action observation") OR ("explicit motor imagery") OR ("graded motor imagery") OR ("guided imagery") OR ("imagined movements") OR ("implicit motor imagery") OR ("laterality recognition") OR ("laterality training") OR ("mental imagery") OR ("mental practice") OR ("mirror box") OR ("mirror therapy") OR ("mirror training") OR ("mirror visual feedback") OR ("motor imagery") OR ("observational learning") OR ("bodily illusion") OR ("eidetic imagery") OR ("guided imagery") OR ("kinesthetic imagery") OR ("mental imagery") OR ("mental movements") OR ("mental practice") OR ("mental rehearsal") OR ("mental simulation") OR ("mental training") OR ("mirror therapy") OR ("motor imagery") OR ("motor observation") OR ("movement observation") OR ("observation training") OR ("perceptual distortion") OR ("visual illusion") OR ("visual imagery"))	**115**

SCOPUS

Search	Query	Results
**S3**	S1 AND S2	**171**
**S2**	TITLE-ABS-KEY ({back ache}) OR TITLE-ABS-KEY ({back dysfunction}) OR TITLE-ABS-KEY ({back pain}) OR TITLE-ABS-KEY ({back strain}) OR TITLE-ABS-KEY ({back syndrome}) OR TITLE-ABS-KEY ({backache}) OR TITLE-ABS-KEY ({cervical pain}) OR TITLE-ABS-KEY ({cervical radiculopathy}) OR TITLE-ABS-KEY ({cervical spondylopathy}) OR TITLE-ABS-KEY ({cervical spondylosis}) OR TITLE-ABS-KEY ({cervicalgia}) OR TITLE-ABS-KEY ({cervicalgia }) OR TITLE-ABS-KEY ({cervicodynia}) OR TITLE-ABS-KEY ({chronic back pain}) OR TITLE-ABS-KEY ({chronic low back pain}) OR TITLE-ABS-KEY ({chronic neck pain}) OR TITLE-ABS-KEY ({discogenic pain}) OR TITLE-ABS-KEY ({dorsalgia}) OR TITLE-ABS-KEY ({low back pain}) OR TITLE-ABS-KEY ({lower back pain}) OR TITLE-ABS-KEY ({lumbago}) OR TITLE-ABS-KEY ({lumbal pain}) OR TITLE-ABS-KEY ({lumbalgia}) OR TITLE-ABS-KEY ({lumbar pain}) OR TITLE-ABS-KEY ({lumbar spine syndrome}) OR TITLE-ABS-KEY ({lumbar spondylosis}) OR TITLE-ABS-KEY ({lumbodynia}) OR TITLE-ABS-KEY ({lumbosacral pain}) OR TITLE-ABS-KEY ({lumbosacral syndrome}) OR TITLE-ABS-KEY ({mechanical low back pain}) OR TITLE-ABS-KEY ({mechanical neck pain}) OR TITLE-ABS-KEY ({neck ache}) OR TITLE-ABS-KEY ({neck pain}) OR TITLE-ABS-KEY ({neck sprain}) OR TITLE-ABS-KEY ({neck symptoms}) OR TITLE-ABS-KEY ({neckache}) OR TITLE-ABS-KEY ({neckpain}) OR TITLE-ABS-KEY ({non specific neck pain}) OR TITLE-ABS-KEY ({non-specific neck pain}) OR TITLE-ABS-KEY ({postural low back pain}) OR TITLE-ABS-KEY ({sacral pain}) OR TITLE-ABS-KEY ({spinal pain}) OR TITLE-ABS-KEY ({spine pain}) OR TITLE-ABS-KEY ({tension neck syndrome}) OR TITLE-ABS-KEY ({thoracic back pain}) OR TITLE-ABS-KEY ({Thoracic pain}) OR TITLE-ABS-KEY ({thoracolumbar pain}) OR TITLE-ABS-KEY ({trapezius myalgia}) OR TITLE-ABS-KEY ({upper back pain}) OR TITLE-ABS-KEY ({vertebral pain}) OR TITLE-ABS-KEY ({whiplash})	**211,997**

**S1**	TITLE-ABS-KEY ({action observation}) OR TITLE-ABS-KEY ({explicit motor imagery}) OR TITLE-ABS-KEY ({graded motor imagery}) OR TITLE-ABS-KEY ({guided imagery}) OR TITLE-ABS-KEY ({imagined movements}) OR TITLE-ABS-KEY ({implicit motor imagery}) OR TITLE-ABS-KEY ({laterality recognition}) OR TITLE-ABS-KEY ({laterality training}) OR TITLE-ABS-KEY ({mental imagery}) OR TITLE-ABS-KEY ({mental practice}) OR TITLE-ABS-KEY ({mirror box}) OR TITLE-ABS-KEY ({mirror therapy}) OR TITLE-ABS-KEY ({mirror training}) OR TITLE-ABS-KEY ({mirror visual feedback}) OR TITLE-ABS-KEY ({motor imagery}) OR TITLE-ABS-KEY ({observational learning}) OR TITLE-ABS-KEY ({bodily illusion}) OR TITLE-ABS-KEY ({eidetic imagery}) OR TITLE-ABS-KEY ({guided imagery}) OR TITLE-ABS-KEY ({kinesthetic imagery}) OR TITLE-ABS-KEY ({mental imagery}) OR TITLE-ABS-KEY ({mental movements}) OR TITLE-ABS-KEY ({mental practice}) OR TITLE-ABS-KEY ({mental rehearsal}) OR TITLE-ABS-KEY ({mental simulation}) OR TITLE-ABS-KEY ({mental training}) OR TITLE-ABS-KEY ({mirror therapy}) OR TITLE-ABS-KEY ({motor imagery}) OR TITLE-ABS-KEY ({motor observation}) OR TITLE-ABS-KEY ({movement observation}) OR TITLE-ABS-KEY ({observation training}) OR TITLE-ABS-KEY ({perceptual distortion}) OR TITLE-ABS-KEY ({visual illusion}) OR TITLE-ABS-KEY ({visual imagery})	**38,278**

Cochrane Library

ID	Search	Hits
**#3**	**#1 AND #2**	**101**
**#2**	("back ache"):ti,ab,kw OR ("back dysfunction"):ti,ab,kw OR ("back pain"):ti,ab,kw OR ("back strain"):ti,ab,kw OR ("back syndrome"):ti,ab,kw OR ("backache"):ti,ab,kw OR ("cervical pain"):ti,ab,kw OR ("cervical radiculopathy"):ti,ab,kw OR ("cervical spondylopathy"):ti,ab,kw OR ("cervical spondylosis"):ti,ab,kw OR ("cervicalgia"):ti,ab,kw OR ("cervicalgia "):ti,ab,kw OR ("cervicodynia"):ti,ab,kw OR ("chronic back pain"):ti,ab,kw OR ("chronic low back pain"):ti,ab,kw OR ("chronic neck pain"):ti,ab,kw OR ("discogenic pain"):ti,ab,kw OR ("dorsalgia"):ti,ab,kw OR ("low back pain"):ti,ab,kw OR ("lower back pain"):ti,ab,kw OR ("lumbago"):ti,ab,kw OR ("lumbal pain"):ti,ab,kw OR ("lumbalgia"):ti,ab,kw OR ("lumbar pain"):ti,ab,kw OR ("lumbar spine syndrome"):ti,ab,kw OR ("lumbar spondylosis"):ti,ab,kw OR ("lumbodynia"):ti,ab,kw OR ("lumbosacral pain"):ti,ab,kw OR ("lumbosacral syndrome"):ti,ab,kw OR ("mechanical low back pain"):ti,ab,kw OR ("mechanical neck pain"):ti,ab,kw OR ("neck ache"):ti,ab,kw OR ("neck pain"):ti,ab,kw OR ("neck sprain"):ti,ab,kw OR ("neck symptoms"):ti,ab,kw OR ("neckache"):ti,ab,kw OR ("neckpain"):ti,ab,kw OR ("non specific neck pain"):ti,ab,kw OR ("non-specific neck pain"):ti,ab,kw OR ("postural low back pain"):ti,ab,kw OR ("sacral pain"):ti,ab,kw OR ("spinal pain"):ti,ab,kw OR ("spine pain"):ti,ab,kw OR ("tension neck syndrome"):ti,ab,kw OR ("thoracic back pain"):ti,ab,kw OR ("thoracic pain"):ti,ab,kw OR ("thoracolumbar pain"):ti,ab,kw OR ("trapezius myalgia"):ti,ab,kw OR ("upper back pain"):ti,ab,kw OR ("vertebral pain"):ti,ab,kw OR ("whiplash"):ti,ab,kw	**31339**
**#1**	("action observation"):ti,ab,kw OR ("explicit motor imagery"):ti,ab,kw OR ("graded motor imagery"):ti,ab,kw OR ("guided imagery"):ti,ab,kw OR ("imagined movements"):ti,ab,kw OR ("implicit motor imagery"):ti,ab,kw OR ("laterality recognition"):ti,ab,kw OR ("laterality training"):ti,ab,kw OR ("mental imagery"):ti,ab,kw OR ("mental practice"):ti,ab,kw OR ("mirror box"):ti,ab,kw OR ("mirror therapy"):ti,ab,kw OR ("mirror training"):ti,ab,kw OR ("mirror visual feedback"):ti,ab,kw OR ("motor imagery"):ti,ab,kw OR ("observational learning"):ti,ab,kw OR ("bodily illusion"):ti,ab,kw OR ("eidetic imagery"):ti,ab,kw OR ("guided imagery"):ti,ab,kw OR ("kinesthetic imagery"):ti,ab,kw OR ("mental imagery"):ti,ab,kw OR ("mental movements"):ti,ab,kw OR ("mental practice"):ti,ab,kw OR ("mental rehearsal"):ti,ab,kw OR ("mental simulation"):ti,ab,kw OR ("mental training"):ti,ab,kw OR ("mirror therapy"):ti,ab,kw OR ("motor imagery"):ti,ab,kw OR ("motor observation"):ti,ab,kw OR ("movement observation"):ti,ab,kw OR ("observation training"):ti,ab,kw OR ("perceptual distortion"):ti,ab,kw OR ("visual illusion"):ti,ab,kw OR ("visual imagery"):ti,ab,kw	**4793**

Pedro

Search	Abstract&Title	Problem	Body Part	Results
#1	Action observation	pain	head or neck	8
#2	Action observation	pain	thoracic spine	0
#3	Action observation	pain	lumbar spine, sacro-iliac joint or pelvis	0
#4	Motor imagery	pain	head or neck	7
#5	Motor imagery	pain	thoracic spine	0
#6	Motor imagery	pain	lumbar spine, sacro-iliac joint or pelvis	5
**Total**	**Duplicates removed**			**16**

Gray Literature:

Search	Search Method	
#1	Turkish Council of Higher Education Thesis Center	2
#2	ProQuest ™ Dissertations & Theses Citation Index	16
#3	Google Search:<br>(“back pain" OR "neck pain") AND ("action observation" OR "motor imagery" OR “mirror therapy”) AND filetype:pdf AND site:.edu<br>270 search results screened	4
